# Artificial Intelligence for Detecting Aortic Arch Calcification on Chest Radiographs: A Systematic Review

**DOI:** 10.3390/diagnostics16020243

**Published:** 2026-01-12

**Authors:** Krzysztof Żerdziński, Julita Janiec, Maja Dreger, Piotr Dudek, Iga Paszkiewicz, Adam Mitręga, Michał Bielówka, Alicja Nawrat, Jakub Kufel, Marcin Rojek

**Affiliations:** 1 Student Scientific Association of Computer Analysis and Artificial Intelligence, Department of Radiology and Nuclear Medicine, Medical University of Silesia, 40-752 Katowice, Poland; kzerdzinskik@gmail.com (K.Ż.); julitajaniec@gmail.com (J.J.); adam.mitrega2306@gmail.com (A.M.); michalbielowka01@gmail.com (M.B.); marcin.arojek@gmail.com (M.R.); 2 Zbigniew Religa Students Department, Biophysics Department, Faculty of Medical Sciences in Zabrze, Medical University of Silesia, 41-808 Zabrze, Poland; 3 Student Scientific Association at the Department of Histology and Cell Pathology, Faculty of Medical Sciences in Zabrze, Medical University of Silesia, 41-808 Zabrze, Poland; 4 Department of Anesthesiology and Intensive Therapy, Branch in Bielsko-Biała, Medical University of Silesia, 43-316 Bielsko-Biała, Poland; igapaszkiewicz.ip@gmail.com; 5 Department of Biophysics, Faculty of Medical Sciences in Zabrze, Medical University of Silesia, 41-808 Zabrze, Poland; 6 Department of Neonatal Intensive Care and Pathology, Faculty of Medical Sciences in Zabrze, Medical University of Silesia, 41-800 Zabrze, Poland; alicja.nawrat@sum.edu.pl; 7 Department of Radiology and Nuclear Medicine, Faculty of Medical Sciences in Katowice, Medical University of Silesia, 40-752 Katowice, Poland; jakubkufel92@gmail.com

**Keywords:** aortic arch calcification, artificial intelligence, cardiovascular risk, chest radiography, convolutional neural networks, deep learning

## Abstract

**Background/Objectives**: Aortic-arch calcification (AAC) is a robust predictor of cardiovascular events often overlooked on routine chest radiographs (CXR). This systematic review aimed to evaluate the diagnostic accuracy of artificial intelligence (AI) models for detecting AAC on CXR and assess their potential for clinical implementation. **Methods**: The review followed PRISMA 2020 guidelines (PROSPERO: CRD420251208627). A search of Embase, PubMed, Scopus, and Web of Science was conducted (Jan 2020–Oct 2025) for studies evaluating AI models detecting AAC in adults. Bias was assessed using QUADAS-2. Due to methodological heterogeneity, a narrative synthesis was performed instead of a meta-analysis. **Results**: Out of 115 records, three retrospective studies (2022–2024) utilizing CNNs across ~2.7 million images were included. Models demonstrated high diagnostic discrimination (AUROC 0.81–0.99), though performance estimates were often attenuated in external cohorts. Pronounced sensitivity–specificity trade-offs occurred: one model achieved 95.9% recall, while another exhibited near-perfect specificity (0.99) despite markedly low sensitivity (0.22). Although the risk of bias was predominantly low, the overall GRADE certainty remained low due to methodological heterogeneity and the absence of cross-sectional imaging reference standards. **Conclusions**: Deep learning-based models reliably detect AAC on routine CXR, offering a scalable tool for opportunistic cardiovascular risk stratification. However, significant heterogeneity in model architectures and validation strategies currently limits broad comparability. Future research requires standardized annotation protocols and external validation to ensure clinical generalizability.

## 1. Introduction

With advancing age, progressive vascular calcification—particularly within the aortic arch—leads to reduced arterial compliance and impaired cardiovascular hemodynamics, contributing to increased cardiac workload and adverse cardiovascular outcomes [1,2,3]. Aortic stiffness, of which aortic arch calcification (AAC) is a measurable manifestation, is an established independent risk factor for major cardiovascular events and mortality [4,5]. Population-based data, including findings from the Rotterdam Study, further indicate a strong association between AAC and the incident of ischemic heart disease [3]. Beyond predicting ischemic heart disease, AAC has been linked to stroke, arterial stiffness, and all-cause mortality. As a marker of systemic arteriosclerosis, it reflects cumulative vascular injury and may precede clinically overt heart disease. Higher AAC grades also independently predict major adverse cardiovascular events in acute coronary syndrome patients undergoing percutaneous coronary intervention and incorporating AAC into clinical prediction models significantly improved risk discrimination and patient reclassification, underscoring its prognostic value even beyond traditional risk factors [6,7,8].

Given this substantial clinical burden, systematic assessment of AAC during routine thoracic imaging is warranted. Chest radiography, representing approximately 40% of more than 3.5 billion imaging studies performed worldwide each year is one of the most accessible, rapid, and cost-effective diagnostic modalities [9]. Furthermore, the growing global demand for imaging exceeds radiologist workforce capacity, resulting in delays in interpretation [10,11]. Under such pressures, small incidental findings—such as early aortic calcifications—are at risk of being overlooked, particularly in high-volume clinical environments.

Artificial intelligence (AI), particularly deep learning (DL)–based methods, has emerged as a scalable solution capable of identifying subtle radiographic features with radiologist-level performance. DL-based algorithms maintain high throughput and diagnostic accuracy regardless of image volume [6,12]. The potential integration of AI into chest radiography workflows offers an opportunity for opportunistic cardiovascular risk stratification through automated AAC detection.

Despite growing interest in artificial intelligence for detecting AAC on chest radiographs, the current evidence base remains methodologically fragmented. Studies differ in AI task definitions (binary classification vs. localization/detection), annotation granularity (presence/absence vs. graded severity), and reference standards (radiologist interpretation vs. computed tomography) [13,14,15]. External validation is inconsistently undertaken, and reporting of key performance characteristics, including operating point selection, calibration, and threshold-dependent measures, is frequently incomplete [13,15]. These inconsistencies limit cross-study comparability and preclude robust meta-analytic synthesis of diagnostic accuracy estimates [16,17].

Therefore, this systematic review aims to synthesize the diagnostic performance of AI algorithms for AAC detection on chest radiographs, appraise methodological quality using established frameworks, and highlight gaps that should be addressed to support future validation and clinical translation.

## 2. Materials and Methods

The systematic review was conducted in accordance with the Preferred Reporting Items for Systematic reviews and Meta-Analyses (PRISMA) 2020 statement guidelines (Supplementary Material S1) and the Cochrane Handbook for Systematic Reviews of Diagnostic Test Accuracy [18,19]. To ensure transparency, the review protocol was registered in the International Prospective Register of Systematic Reviews (PROSPERO) before data extraction, on 12 November 2025 (CRD420251208627).

### 2.1. Eligibility Criteria

We included original studies evaluating artificial intelligence (AI) models (machine learning or deep learning, including CNNs and CAD systems) developed or validated for automatic detection, segmentation, or classification of AAC on posteroanterior or anteroposterior chest X-ray in adult humans (≥18 years). Eligible designs were diagnostic-accuracy studies (cross-sectional, cohort, case–control, reader or technical validation studies) using clinical or public datasets from any healthcare setting, provided that image-level labels for AAC and a clearly defined reference standard were available. Model-development papers were included only if they reported performance on an independent validation or test set using diagnostic metrics (e.g., sensitivity, specificity, AUROC, PR-AUC) and/or segmentation metrics (e.g., Dice/F1, IoU, mAP).

Comparators, when present, could include human readers (radiologists or clinicians), routine clinical interpretation, or imaging-based standards such as computed tomography or expert consensus on CXR. A formal comparator was not required if the reference standard for AAC was clearly defined.

We excluded studies in paediatric or animal populations, phantom or simulation studies, non-imaging work, and studies not explicitly targeting AAC on CXR (e.g., only coronary or abdominal aortic calcification, non-radiographic modalities, CT-only models, lateral-only radiographs, fluoroscopy, or tomosynthesis). We also excluded rule-based algorithms without a machine-learning component, randomised workflow or impact trials without image-level diagnostic-accuracy results, case reports, small case series, reviews, editorials, letters, protocols, theses without full data, and conference abstracts without full results. Only peer-reviewed, English-language articles published between 1 January 2020 and 20 October 2025 were considered.

### 2.2. Information Sources

We systematically searched four electronic bibliographic databases: Embase.com, PubMed/MEDLINE, Scopus, and Web of Science. The searches were last performed on 20 October 2025 and were limited to peer-reviewed articles published in English. No restrictions were applied regarding country, clinical setting, or study design beyond the eligibility criteria. No additional sources (e.g., trial registries, preprint servers, conference proceedings, or manual searches of reference lists) were searched in a systematic manner. No search updates were performed after the initial run.

### 2.3. Search Strategy

We developed database-specific search strategies for PubMed/MEDLINE, Embase, Web of Science, and Scopus by combining controlled vocabulary (MeSH, Emtree) and Title/Abstract/Key Words terms for three core concepts: (1) aorta/aortic arch and vascular calcification, (2) chest radiography or chest X-ray, and (3) artificial intelligence, machine learning, and deep learning (including common architectures and algorithms such as convolutional neural networks, U-Net, transformers, ResNet, DenseNet, EfficientNet, VGG, SegNet, XGBoost, random forests, and support vector machines).

For each database, the search string was adapted to its syntax and indexing structure, using field tags (e.g., Title/Abstract, TITLE-ABS-KEY, TS) and database-specific subject headings (e.g., Aorta, Vascular Calcification, Radiography, Thoracic, Artificial Intelligence, Machine Learning, Deep Learning). We did not apply filters for study design to maximise sensitivity. Searches were restricted to English-language publications between 1 January 2020 and 20 October 2025, in line with the eligibility criteria. The complete search strategies for all databases (PubMed, Embase, Web of Science, Scopus) are provided in the Supplementary Material S2.

### 2.4. Study Selection and Screening

All records retrieved from the database searches were imported into a reference manager for deduplication and then uploaded to a web-based screening tool (Rayyan web-based platform; accessed on 20 October 2025). Three reviewers independently screened titles and abstracts against the predefined eligibility criteria. Full texts were then assessed independently by the same three reviewers. Disagreements at any stage were resolved by discussion and, if necessary, by consultation with a fourth reviewer. No automation tools were used for screening.

### 2.5. Data Collection Process

Data were extracted from the published articles and reports using a predefined data-extraction form aligned with the eligibility criteria and planned outcomes. Two reviewers independently extracted data from each included study, and any discrepancies were resolved by the third author. We did not contact study authors to obtain missing or unpublished data.

### 2.6. Data Items

Data from each included study were extracted using a predefined data-extraction form based on the PROSPERO protocol and the planned outcomes of this review. Any discrepancies were first discussed between the two reviewers, and unresolved disagreements were arbitrated by a third author.

For each study, we collected information on basic study identifiers (first author, year of publication), study design and setting, population characteristics, imaging protocol (type of chest X-ray and acquisition details), AI model characteristics (type of algorithm, architecture, training and validation strategy), reference standard, and reported performance metrics (e.g., sensitivity, specificity, area under the ROC curve, and other classification or segmentation measures when available). When relevant information was unclear or not reported in the article, it was recorded as “not reported” and no additional assumptions were made.

### 2.7. Risk of Bias & Applicability

The risk of bias and applicability concerns were evaluated using the Quality Assessment of Diagnostic Accuracy Studies 2 (QUADAS-2) tool [20]. This assessment encompasses four key domains: patient selection, index test, reference standard, and flow and timing. The evaluation was conducted independently by two reviewers, with any discrepancies resolved through discussion or arbitration by a third author.

Specific signaling questions were tailored to address the nuances of artificial intelligence research in this context. In the patient selection domain, studies utilizing case–control designs or artificially balanced datasets were classified as high risk. Regarding the index test, the risk of bias was deemed high if decision thresholds were not pre-specified or were optimized on the test set, indicating potential data leakage. A low-risk reference standard was defined as either computed tomography or a consensus of at least two expert radiologists.

The quality assessment results are summarized in tables and figures. Studies exhibiting high risk of bias in one or more domains were not excluded from the review. However, their potential influence on the reliability of diagnostic accuracy estimates has been incorporated into the synthesis and interpretation of the results.

The certainty of evidence for the main diagnostic accuracy outcome was assessed using the GRADE approach for diagnostic tests. Two reviewers (J.J. and K.Ż.) independently performed the assessment, and any disagreements were resolved by a third reviewer (M.R.). We considered the standard GRADE domains for diagnostic test accuracy, including risk of bias, inconsistency, indirectness, and imprecision, and rated the overall certainty accordingly.

### 2.8. Effect Measures

The primary effect measures for this review were diagnostic accuracy metrics for the detection of AAC on chest X-ray. We extracted sensitivity and specificity, and other performance metrics as reported by study authors. We attempted to extract or reconstruct 2 × 2 tables (true positives, false positives, true negatives, false negatives), but this was feasible for only one study. Therefore, we did not compute additional diagnostic accuracy estimates or confidence intervals beyond what was reported. As secondary measures, we collected AUROC, PR-AUC, accuracy, F1-score, positive and negative predictive values, and likelihood ratios, when reported. For segmentation or localisation tasks, we extracted segmentation metrics such as Dice/F1 coefficient, intersection-over-union (IoU), and mean average precision at a defined IoU threshold (mAP), where available.

### 2.9. Synthesis Methods

We did not perform a formal quantitative meta-analysis. Instead, study results were summarised using a structured narrative synthesis. For each included study, we presented the main characteristics (population, imaging protocol, AI model, reference standard, and validation strategy) together with the reported diagnostic performance metrics in summary tables.

Within the narrative synthesis, we compared studies according to (1) the type of AI task (binary detection vs. segmentation/localisation), (2) the type of reference standard (expert reader consensus vs. CT-based or other standards), (3) the validation level (internal vs. external validation), and (4) the presence and type of comparator (e.g., human readers or usual clinical interpretation). For diagnostic-accuracy outcomes, we described ranges and patterns of sensitivity, specificity, AUROC, and other reported metrics across studies, with particular emphasis on performance in externally validated models. For segmentation tasks, we summarised reported Dice/F1, IoU, and related measures.

Findings from the risk of bias and applicability assessment were integrated into the interpretation of results, with explicit comment on whether studies at higher risk of bias or with limited external validity showed systematically different performance estimates. Where multiple models or operating points were reported in a single study, we highlighted in the narrative the model and threshold prespecified by the authors as primary or, if not specified, the model and operating point evaluated on an independent test set. We reported confidence intervals only when provided in the original publications.

## 3. Results

### 3.1. Study Selection Results

**Figure 1:**
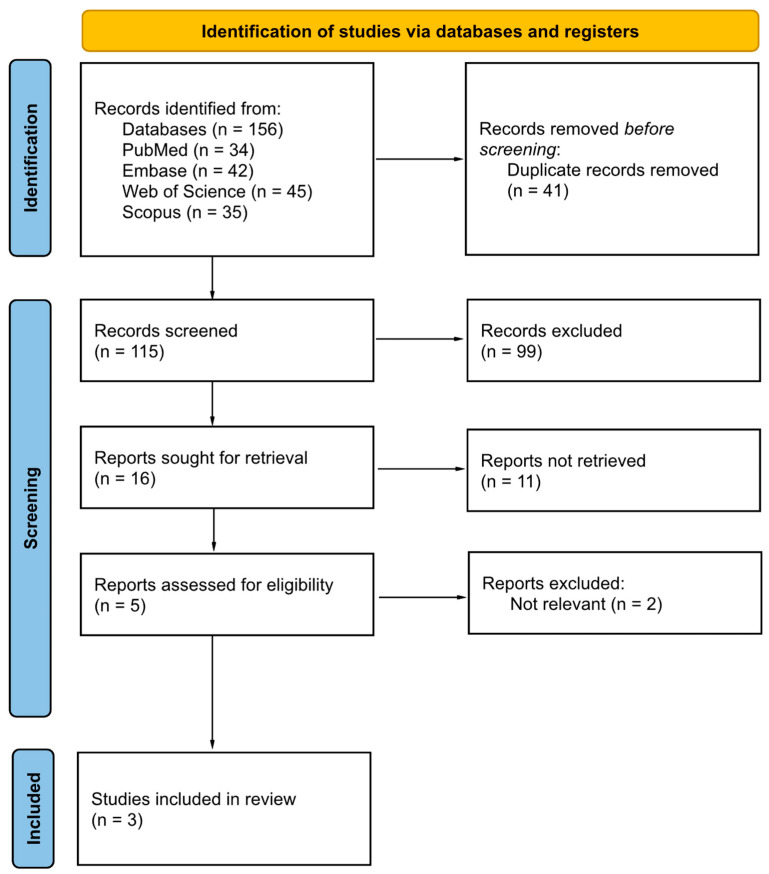
PRISMA flow diagram of study screening and selection [18].

A total of 156 records were identified through database searching (PubMed n = 34, Embase n = 42, Web of Science n = 45, Scopus n = 35). After removing 41 duplicates, 115 records were screened, and 99 were excluded based on title and abstract. Full texts were sought for 16 reports, of which 11 could not be retrieved. 5 reports were assessed for eligibility, and 2 were excluded as not relevant. Finally, 3 studies were included in the review. The selection process is summarized in the PRISMA flow diagram (Figure 1) [18].

**Figure 2:**
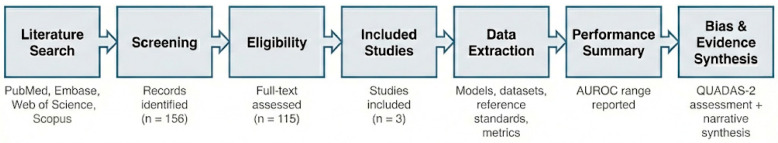
Analytical pipeline of the systematic review.

Figure 2 summarizes the analytical pipeline used for study selection, data extraction, and evidence synthesis in this systematic review.

### 3.2. Study Characteristics

Three retrospective studies published between 2022 and 2024 were included in the review. The studies were conducted in Taiwan [21], China [22], and the United Kingdom [9]. One study focused exclusively on the detection of AAC [21], while the remaining two evaluated AAC detection as part of comprehensive multi-label AI systems for chest X-ray interpretation [9,22].

The cumulative sample size was ~2,768,923, leveraging large-scale clinical datasets and public repositories (e.g., VinDr-CXR). The developed models were based on CNNs, utilizing architectures such as DenseNet, EfficientNet, and ensemble methods. Reference standards varied across studies, including radiologist consensus, physician-labeled public datasets, and report/NLP-derived labels, with a radiologist-consensus subset used in one study. The detailed characteristics of the included studies are summarized in Table 1.

### 3.3. Risk of Bias in Studies

The risk of bias assessment using the QUADAS-2 tool revealed heterogeneity in the methodological quality of the included studies. Two studies [9,22] were rated as having low risk of bias across all four domains: patient selection, index test, reference standard, and flow and timing. This reflects the use of consecutive or random patient selection, pre-specified thresholds, and appropriate reference standards in these large-scale studies.

However, the study by Chao et al. [21] was assessed as having high risk of bias overall. Specifically, high risk was noted in the “patient selection” and “index test” domains, suggesting potential issues with the study design (e.g., case–control sampling) or lack of pre-specified decision thresholds. Additionally, the “reference standard” domain for this study was rated as having some concerns, which differs from the clear low-risk assessment in the other two studies. 

**Figure 3:**
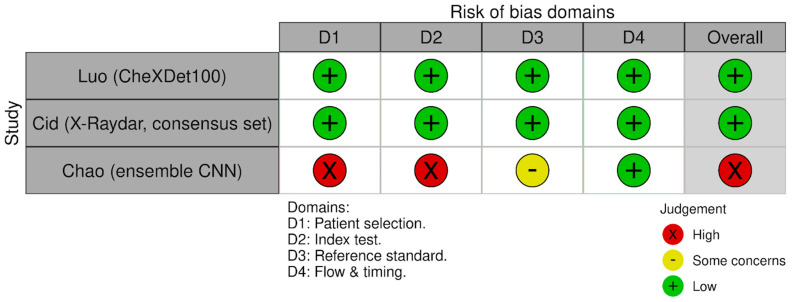
Study-level risk of bias assessment using the QUADAS-2 tool.

Despite these differences in methodological quality, applicability concerns were deemed low across all domains for all three included studies. The risk of bias summary is presented in Figure 3.

### 3.4. Individual Study Findings and Narrative Synthesis

Three studies met the inclusion criteria and were synthesized narratively. Owing to the limited number of eligible studies and substantial methodological and clinical heterogeneity, a quantitative meta-analysis was not performed. The included investigations differed markedly in study design, dataset composition and scale, annotation strategies and reference standards, model architectures, and validation approaches, precluding meaningful statistical pooling of diagnostic performance metrics. Confidence intervals and consistent operating thresholds were not uniformly available across studies, and only one study provided large-scale external validation with narrow CIs. Consequently, results are presented as a structured narrative synthesis focused on AAC detection, integrating study-level findings with cross-study comparison of performance ranges, validation strategies, and sources of uncertainty.

Chao et al. [21] developed an ensemble deep-learning system for binary detection of AAC on chest radiographs, combining four convolutional neural networks trained with focal loss to address marked class imbalance. In the primary test cohort (n = 233), the ensemble achieved an AUC of 0.85, with high recall for AAC (95.9%) and moderate specificity (73.5%), reflecting a tendency toward sensitive detection at the expense of false positives. External evaluation on a small pre-end-stage kidney disease cohort (n = 24) yielded a comparable AUC of 0.86, although the limited sample size and absence of confidence intervals substantially constrain inference regarding generalizability and precision.

Luo et al. [22] evaluated both a multi-label classification model (CheXNet) and a lesion-level detection model (CheXDet) for aortic calcification as part of a broader chest radiograph framework. Using a large internal multicenter dataset (34,501 images), both approaches demonstrated high discrimination for AAC, with AUCs approaching 0.98–0.99 in a radiologist-comparison subset. External validation on the PadChest dataset (24,536 images) showed consistently high performance across model variants, with AUCs ranging from 0.81 to 0.87 and narrow confidence intervals, supporting robustness at scale. However, reference standards relied on radiologist consensus and report-derived labels rather than cross-sectional imaging, introducing potential label noise and indirectness specific to aortic arch assessment.

Dicente Cid et al. [9] reported AAC detection as one component of the large-scale X-Raydar system, trained predominantly on labels derived from natural language processing of radiology reports and evaluated across multiple datasets. Diagnostic performance varied substantially by reference standard and cohort: AUC reached 0.91 in an auto-labelled dataset, decreased to 0.87 on external MIMIC-CXR data, and was lower (0.81) in a radiologist-consensus test set enriched for AAC. At a fixed operating threshold in the consensus cohort, specificity was very high (0.99) but sensitivity was low (0.22), indicating a strong rule-in but poor rule-out profile. These findings highlight pronounced sensitivity–specificity trade-offs and underscore the influence of label derivation and dataset composition on reported accuracy.

Across the three included studies, diagnostic discrimination for AAC on chest radiographs was consistently moderate to high, with reported AUC values ranging from 0.81 to 0.99, depending on dataset composition, reference standard, and evaluation setting. Higher AUCs were generally observed in internal validations and in cohorts enriched for clearly defined AAC cases, whereas performance estimates were attenuated in external or consensus-based evaluations, suggesting sensitivity to spectrum effects and dataset shift. Notably, the apparent upper range of performance was largely driven by internal comparisons or auto-labelled datasets, while externally validated results clustered within a narrower and more conservative range.

Substantial variability was observed in sensitivity and specificity trade-offs across studies and operating thresholds. Chao et al. [21] reported high sensitivity with moderate specificity, favoring case detection at the cost of false positives, whereas Dicente Cid et al. [9] demonstrated the opposite pattern in a radiologist-consensus cohort, with very high specificity but markedly low sensitivity, consistent with a rule-in rather than screening application. These divergent profiles underscore that reported accuracy metrics are highly dependent on threshold selection and intended clinical use, and that no uniform operating point can be inferred across studies.

Reference standards differed meaningfully between investigations and exerted a clear influence on reported performance. Studies relying on radiologist consensus or report-derived labels, including NLP-based annotations, are inherently susceptible to label noise and verification bias, particularly in the absence of cross-sectional imaging confirmation. Performance estimates were systematically higher in auto-labelled or report-based datasets and lower when restricted to manually adjudicated consensus sets, indicating that apparent accuracy may partly reflect differences in ground truth definition rather than true model capability.

External validation was inconsistently implemented and varied in rigor. One study provided large-scale external testing with narrow confidence intervals, supporting technical robustness, whereas another relied on a very small external cohort, limiting conclusions regarding generalizability. Collectively, the evidence suggests that while AI-based AAC detection on chest radiographs is feasible, its generalizability across populations, institutions, and labeling paradigms remains uncertain, and reported performance should be interpreted in the context of dataset shift, reference standard heterogeneity, and intended clinical deployment.

### 3.5. Certainty of Evidence (GRADE)

Overall certainty of evidence for AI-based detection of AAC on chest radiographs was low, despite generally moderate-to-high discrimination across datasets [9,21,22]. The main drivers of downgrading were serious risk of bias and serious indirectness, with imprecision affecting settings with small external tests or incomplete uncertainty reporting [21], and inconsistency reflecting heterogeneity in labeling paradigms and evaluation cohorts [9].

Risk of bias was judged seriously. All studies were retrospective and susceptible to selection and spectrum effects due to dataset construction and case mix [9,21,22]. Reporting of annotation procedures and adjudication was incomplete in places, limiting confidence in label reliability and comparability [9,21]. In addition, pipelines relying on report-derived labels, including NLP-assisted labeling, may inflate apparent performance when evaluation uses similarly derived labels [9].

Indirectness was judged seriously, because reference standards differed and none provided a uniform cross-sectional imaging confirmation for AAC. Luo et al. [22] used radiologist consensus supported by report review internally and physician-labeled PadChest externally, which may introduce label noise for AAC. Dicente Cid et al. [9] showed materially different AUCs across auto-labelled, external, and radiologist-consensus sets, indicating that the “ground truth” definition and cohort enrichment strongly influence observed accuracy. Chao et al. [21] similarly relied on radiologist/specialist labeling without CT confirmation, limiting comparability across settings.

Imprecision varied. Chao et al. [21] reported external testing in a very small cohort (n = 24) without confidence intervals, substantially limiting precision and generalizability. In contrast, Luo et al. [22] provided large-scale external validation on PadChest with narrow 95% confidence intervals. Dicente Cid et al. [9] reported confidence intervals across large datasets, yet the operating-point results in the radiologist-consensus cohort revealed very low sensitivity at high specificity, increasing uncertainty regarding screening utility despite acceptable AUC.

Publication bias could not be assessed given the small evidence base. Overall, the body of evidence supports low certainty, with very low certainty in settings driven by small external samples or missing uncertainty estimates [21], and comparatively stronger but still limited certainty where large external validation with confidence intervals was available under non-CT reference standards [22].

## 4. Discussion

### 4.1. Principal Findings

In this systematic review, three studies evaluating the use of artificial intelligence for detecting AAC on chest radiographs were identified. Although the number of available studies is limited, current evidence indicates that AI models achieve good to very good diagnostic performance, with the highest effectiveness observed in models trained on large and diverse datasets.

However, model performance remains strongly dependent on the underlying architecture, annotation quality, and validation strategy. Methodological assessment revealed a generally low risk of bias, although one study showed notable limitations related to sample selection and the determination of decision thresholds.

The overall certainty of the evidence was rated as low, primarily due to variability in reference standards and heterogeneity across study designs. Despite these constraints, the findings suggest that AI may support the detection of AAC and serve as a useful tool for opportunistic cardiovascular risk stratification.

### 4.2. Positioning of Findings Within the Broader Literature

AI-based detection of vascular calcification is most mature for coronary artery calcification (CAC) on computed tomography. Large studies and systematic reviews report high diagnostic accuracy, near-perfect agreement with manual Agatston scoring, and consistent prognostic utility on both ECG-gated and non-gated chest CT [23,24,25]. This evidence translated into clinical guidance, with endorsement of AI-enabled CAC quantification from routine chest CT as a scalable strategy for opportunistic cardiovascular prevention [26].

Evidence for AI-based thoracic aortic calcification on CT is less established. Although automated quantification shows excellent agreement with manual scoring, its incremental prognostic value beyond CAC appears limited in asymptomatic screening populations, restricting clinical uptake compared with CAC-focused tools [27].

Chest radiography represents a different and more constrained context. AAC visible on chest X-ray has long been associated with coronary heart disease, ischemic stroke, and mortality [3,28], and higher AAC grades predict adverse outcomes in acute coronary syndrome cohorts, improving risk reclassification when added to conventional risk scores [8,29]. However, compared with CT, diagnostic validity is moderate, with high specificity but low sensitivity for severe coronary calcification, susceptibility to projection and image-quality effects, and frequent under-reporting in routine reporting [30,31].

Contemporary AI for chest radiographs has primarily targeted pulmonary and broad cardiac findings. In large multi-label systems, vascular calcification is often absent or captured under non-specific labels derived from report-based annotations, and performance for vascular/mediastinal findings tends to lag behind pulmonary abnormalities [9,32]. Crucially, before this review, no systematic synthesis specifically addressed AI models dedicated to AAC detection on chest radiographs.

Accordingly, this review bridges three evidence streams: the prognostic relevance of AAC on CXR, the implementation-ready maturity of AI CAC scoring on CT, and the current limitations of general-purpose CXR AI for vascular findings. Although only three eligible studies were identified, the available evidence suggests that deep learning models can achieve fair-to-excellent discrimination for AAC detection in large datasets, while heterogeneity in task definitions, annotation strategies, and validation approaches still limits cross-study comparability. Overall, AAC-on-CXR AI remains a promising opportunistic marker, but it is not yet positioned to match the clinical readiness of CT-based CAC quantification.

### 4.3. Limitations

This systematic review has several limitations. First, the evidence base is very small: only three eligible studies were identified, which precluded meta-analysis and limits the strength and generalizability of conclusions.

Second, substantial heterogeneity across studies (AI task definitions, annotation granularity, reference standards, validation strategies, and reported metrics) prevented meaningful pooling and restricted direct comparability. In particular, incomplete reporting of threshold-dependent measures and 2 × 2 data limited the ability to synthesize sensitivity and specificity.

Third, the search strategy was intentionally restricted to peer-reviewed journal articles. While this approach was chosen to prioritize methodological rigor, transparency, and comparability of evidence, it may have resulted in the omission of relevant studies reported exclusively as preprints, conference abstracts, or posters. Such sources were excluded because they frequently lack essential methodological details and complete performance reporting required for reliable assessment of diagnostic accuracy. Nonetheless, this restriction may contribute to publication bias and should be considered when interpreting the findings.

Fourth, all included studies were retrospective, with no prospective or workflow-integrated evaluations, so real-world performance and clinical impact remain uncertain. Reference standards also varied and were largely based on radiologist interpretation or report-derived labels rather than CT, introducing potential misclassification and contributing to low certainty of evidence.

Finally, external validation was inconsistent, and publication bias cannot be excluded given the small number of studies and predominance of positive results. Overall, current findings should be considered preliminary until confirmed by standardized methods, robust external validation, and prospective clinical studies.

### 4.4. Future Research and Implementation Priorities

Future work should focus on progressing AAC-on-CXR algorithms from retrospective validation toward clinically deployable decision support, aligned with accepted evaluation frameworks for imaging AI. Across guidance, robust generalizability is a prerequisite: external validation on independent datasets is required, and internal validation alone is insufficient for adoption [15,33,34]. For AAC specifically, validation should be deliberately stratified by projection and acquisition context, because vascular/mediastinal targets are disproportionately affected by AP/portable films, positioning, and anatomical overlap, which also explains their lower sensitivity compared with pulmonary findings in real-world CXR AI deployments [32,34]. Prospective and workflow-integrated evaluations should follow, measuring safety, efficiency, and reader–AI interaction rather than accuracy alone [35,36].

A second priority is improving reference standards and annotation. Transparent ground-truth definition is repeatedly emphasized for trustworthy translation [13]. For AAC-on-CXR, CT correlation is the most defensible reference standard when feasible, while radiologist consensus remains acceptable for large-scale labeling provided that discordant or equivocal cases are adjudicated and interrater agreement is reported [30,37]. Studies should converge on clinically interpretable targets, ideally graded AAC severity alongside binary labels, and consider lesion-level localization when the intended output is “detection” rather than coarse classification.

Third, evaluation and reporting should be explicitly clinical. Guidance stresses prespecified operating points, calibration assessment, and complete threshold-dependent reporting, since post hoc threshold optimization and poor calibration can undermine safety [13,15,38]. Minimum requirements for AAC detection should include reconstructable 2 × 2 data, sensitivity and specificity at prespecified thresholds, and calibration. Because AAC is an opportunistic risk marker, decision-analytic approaches (e.g., net benefit) are also relevant to show whether AI output meaningfully improves downstream actions beyond routine reporting [38,39].

Implementation should be framed around a realistic pathway: structured-reporting support and standardized follow-up triggers (e.g., prompting formal cardiovascular risk assessment) rather than autonomous “management recommendations” [40,41]. Finally, deployment requires life-cycle monitoring for dataset shift and fairness across demographic subgroups, and explicit accountability for incidental findings [42].

## 5. Conclusions

The review confirms that deep learning–based systems are capable of reliably identifying AAC on conventional chest radiographs, achieving levels of accuracy comparable to those reported in other domains of thoracic imaging. High AUROC values observed in large, multi-center studies indicate that the use of extensive training datasets and consistent annotation protocols improves the generalizability of AI models. At the same time, weaker performance in detection models suggests that tasks requiring precise localization of calcified lesions are more sensitive to annotation quality and to the structural complexity of the calcifications themselves.

Another challenge is the substantial heterogeneity across studies—including differences in populations (e.g., dialysis cohorts vs. general population, multi-center datasets), AI task types (classification vs. detection), performance metrics, and validation strategies. This variability prevents formal meta-analysis and limits the ability to perform direct comparisons. Future research would benefit from standardized annotation schemes, harmonized evaluation metrics, and the consistent use of external validation in populations with diverse cardiovascular risk profiles.

From a clinical perspective, the findings are promising. AI systems have the potential to enhance the diagnostic utility of routine chest radiography, which already represents a large proportion of imaging performed worldwide. Automated detection of AAC could support early identification of individuals at elevated cardiovascular risk, particularly in high-volume settings where subtle calcifications may be overlooked. However, implementation in clinical practice requires further investigation into workflow impact, model robustness across imaging equipment and acquisition protocols, and comparative performance relative to expert radiologist interpretation.

## Figures and Tables

**Table 1:** Summary of the characteristics and methodology of the retrospective studies included in the review.

Author (Year)	Country	Study Design	Dataset Source	Total Sample Size (Images)	Validation	AI Task Related to AAC	AI Model Architecture	Reference Standard
Chao et al. (2023) [21]	Taiwan (hospital); Vietnam (public dataset)	Retrospective model development and validation	VinDr-CXR (public) + NTUH cohorts (older adults; pre-ESKD)	11,165 (10,767 VinDr + 339 NTUH older adults + 59 NTUH pre-ESKD)	Internal test (n = 233) + external NTUH cohorts (older adults; pre-ESKD test n = 24)	Binary CXR classification: AAC present vs. absent	Ensemble of 4 CNNs (VGG16, Xception, DenseNet121, MobileNetV2), focal loss	Radiologist-labeled ground truth (VinDr annotations; NTUH radiologist/specialist AAC labeling)
Luo et al. (2022) [22]	China (Hong Kong/Shenzhen) + multicenter public datasets	Retrospective multicenter development and external validation	Internal DS1 + external PadChest (AAC); (NIH Google not for AAC)	34,501 (DS1) + 24,536 (PadChest AAC test)	Internal test (DS1 n = 2922) + external (PadChest n = 24,536) + radiologist subset (n = 496)	Radiograph-level classification: aortic calcification present vs. absent	CheXNet (DenseNet-121 classifier) and CheXDet (EfficientNet + BiFPN two-stage detector; bobs-trained, used for image-level AUC)	DS1: 2 radiologists + report consensus (bbox adjudicated by senior radiologist); PadChest: radiograph-level labels by trained physicians
Dicente Cid et al. (2024) [9]	UK	Retrospective, multicentre	6 UK hospitals (X-Raydar dataset) + external MIMIC-CXR; consensus set	2,513,546	Internal + external (MIMIC-CXR); separate radiologist consensus test set	Multi-label CXR classification incl. AAC	X-Raydar (DenseNet-121–based MLC) + X-Raydar-NLP for labels	Auto-labels from NLP (train/auto-labelled) + radiologist consensus on consensus set

## Data Availability

The data used during this study are available from the corresponding author upon reasonable request.

## Supplementary Materials

The following supporting information can be downloaded at: https://www.mdpi.com/article/10.3390/diagnostics16020243/s1, File S1: PRISMA 2020 Checklist. File S2: Search strategies for all databases.

## Abbreviations

AAC	Aortic arch calcification
CXR	Chest radiographs
CNN	Convolutional neural network
PRISMA	The Preferred Reporting Items for Systematic reviews and Meta-Analyses
PROSPERO	International Prospective Register of Systematic Reviews
QUADAS-2	Quality Assessment of Diagnostic Accuracy Studies 2
AUROC	Area under the receiver operating characteristic curve
PR-AUC	Precision–recall area under the curve
IoU	Intersection-over-union
CAC	Coronary artery calcification
