# Supplementary material for: Artificial Intelligence for Detecting Aortic Arch Calcification on Chest Radiographs: A Systematic Review
Source: Diagnostics (Basel). 2026 Jan 12;16(2):243. doi: 10.3390/diagnostics16020243 (PMC12839748; doi:10.3390/diagnostics16020243)
Supplement: Supplementary file 1 [file diagnostics-16-00243-s001.zip › File S2_Search strategies for all databases.pdf]

PubMed:

(
(aort*[Title/Abstract] OR Aorta[MeSH Terms] OR Aorta, Thoracic[MeSH Terms])
AND
(calcif*[Title/Abstract] OR Vascular Calcification[MeSH Terms] OR Calcinosis[MeSH Terms])
)
AND
(
(
(X-Rays[MeSH Terms] OR Radiography[MeSH Terms]
OR imaging[Title/Abstract] OR x-ray[Title/Abstract] OR radiograph*[Title/Abstract] OR scan*[Title/Abstract]
)
AND
(chest[Title/Abstract] OR thora*[Title/Abstract]))
OR
Radiography, Thoracic[MeSH Terms] OR Mass Chest X-Ray[MeSH Terms]
)
AND
(
artificial intelligence[MeSH Terms] OR machine learning[MeSH Terms] OR deep learning[MeSH Terms]
OR Neural Networks, Computer[MeSH Terms] OR Convolutional Neural Networks[MeSH Terms]
OR Generative Artificial Intelligence[MeSH Terms]
OR artificial intelligence[Title/Abstract] OR machine learning[Title/Abstract] OR deep learning[Title/Abstract] OR neural network[Title/Abstract]
OR AI[Title/Abstract] OR ML[Title/Abstract] OR DL[Title/Abstract] OR CNN[Title/Abstract] OR U-Net[Title/Abstract] OR vision transformer[Title/Abstract] OR ViT[Title/Abstract]
OR ResNet[Title/Abstract] OR DenseNet[Title/Abstract] OR EfficientNet[Title/Abstract] OR VGG[Title/Abstract] OR SegNet[Title/Abstract] OR XGBoost[Title/Abstract]
OR random forest[Title/Abstract] OR support vector machine[Title/Abstract] OR SVM[Title/Abstract] OR Computer-Assisted[Title/Abstract]
OR transformer network[Title/Abstract] OR unsupervised learning[Title/Abstract] OR supervised learning[Title/Abstract] OR transfer learning[Title/Abstract]
)

Embase:

(
((aort*:ti,ab,kw OR Aorta/exp) AND (calcif*:ti,ab,kw OR blood vessel calcification/exp))
OR
(aort* NEAR/3 calcif*):ti,ab,kw
)
AND
(
(
(imaging OR "x ray" OR radiograph* OR scan*):ti,ab,kw
OR
x ray/exp
OR
radiodiagnosis/exp

OR
radiography/exp
OR
thorax radiography/exp)
AND
(chest OR thora*):ti,ab,kw
)
)
AND
(
artificial intelligence/exp OR machine learning/exp OR deep learning/exp OR Convolutional Neural Networks/exp
OR
(artificial intelligence OR machine learning OR deep learning OR neural network OR AI OR ML OR DL OR CNN
OR U-Net OR "vision transformer" OR ViT OR ResNet OR DenseNet OR EfficientNet OR VGG OR SegNet
OR XGBoost OR "random forest" OR "support vector machine" OR SVM OR "Computer-Assisted"
OR "transformer network" OR "unsupervised learning" OR "supervised learning" OR "transfer learning"
):ti,ab,kw
)

((A AND B) OR C) AND ((D OR E OR F OR G OR H) AND I) AND J

Web of Science:

(
TS=(aort* AND calcif*)
)
AND
(
TS=(imaging OR "x-ray" OR radiograph* OR scan*)
AND
TS=(chest OR thora*)
)
AND
TS=(artificial intelligence OR "machine learning" OR "deep learning" OR "neural network"
OR AI OR ML OR DL OR CNN OR "U-Net" OR "vision transformer" OR ViT OR ResNet
OR DenseNet OR EfficientNet OR VGG OR SegNet OR XGBoost OR "random forest"
OR "support vector machine" OR SVM OR "computer-assisted" OR "transformer network"
OR "unsupervised learning" OR "supervised learning" OR "transfer learning")

Scopus:

(
TITLE-ABS-KEY(aort* AND calcif*)
)
AND
(
TITLE-ABS-KEY(imaging OR "x-ray" OR radiograph* OR scan*)

AND
TITLE-ABS-KEY(chest OR thora*)
)
AND
TITLE-ABS-KEY(artificial intelligence OR "machine learning" OR "deep learning" OR "neural network"
OR AI OR ML OR DL OR CNN OR "U-Net" OR "vision transformer" OR ViT OR ResNet
OR DenseNet OR EfficientNet OR VGG OR SegNet OR XGBoost OR "random forest"
OR "support vector machine" OR SVM OR "computer-assisted" OR "transformer network"
OR "unsupervised learning" OR "supervised learning" OR "transfer learning")
